# Conformational changes and loose packing promote *E. coli *Tryptophanase cold lability

**DOI:** 10.1186/1472-6807-9-65

**Published:** 2009-10-08

**Authors:** Anna Kogan, Garik Y Gdalevsky, Rivka Cohen-Luria, Yehuda Goldgur, Robert S Phillips, Abraham H Parola, Orna Almog

**Affiliations:** 1 Department of Chemistry, Ben-Gurion University of the Negev, POB 653, Beer-Sheva 84105, Israel; 2 Departments of Chemistry and Biochemistry and Molecular Biology, University of Georgia, Athens, GA 30602, USA; 3 Department of Clinical Biochemistry, Faculty of Health Sciences, Ben-Gurion University, Beer-Sheva 84105, Israel

## Abstract

**Background:**

Oligomeric enzymes can undergo a reversible loss of activity at low temperatures. One such enzyme is tryptophanase (Trpase) from *Escherichia coli*. Trpase is a pyridoxal phosphate (PLP)-dependent tetrameric enzyme with a Mw of 210 kD. PLP is covalently bound through an enamine bond to Lys270 at the active site. The incubation of holo *E. coli *Trpases at 2°C for 20 h results in breaking this enamine bond and PLP release, as well as a reversible loss of activity and dissociation into dimers. This sequence of events is termed cold lability and its understanding bears relevance to protein stability and shelf life.

**Results:**

We studied the reversible cold lability of *E. coli *Trpase and its Y74F, C298S and W330F mutants. In contrast to the holo *E. coli *Trpase all apo forms of Trpase dissociated into dimers already at 25°C and even further upon cooling to 2°C. The crystal structures of the two mutants, Y74F and C298S in their apo form were determined at 1.9Å resolution. These apo mutants were found in an open conformation compared to the closed conformation found for *P. vulgaris *in its holo form. This conformational change is further supported by a high pressure study.

**Conclusion:**

We suggest that cold lability of *E. coli *Trpases is primarily affected by PLP release. The enhanced loss of activity of the three mutants is presumably due to the reduced size of the side chain of the amino acids. This prevents the tight assembly of the active tetramer, making it more susceptible to the cold driven changes in hydrophobic interactions which facilitate PLP release. The hydrophobic interactions along the non catalytic interface overshadow the effect of point mutations and may account for the differences in the dissociation of *E. coli *Trpase to dimers and *P. vulgaris *Trpase to monomers.

## Background

Enzymes can undergo a reversible loss of activity at low temperatures, a process that is termed cold inactivation [1,2]. This phenomenon is found in a widespread variety of oligomeric enzymes, e.g, pyrophosphatase [3], pyruvate carboxylase [4], alcohol dehydrogenase [5], as well as PLP-dependent enzymes such as glutamic acid decarboxylase [6] and tryptophanase (Trpase) [7-9]. It has been proposed that cooling evokes major changes in hydrophobic interactions which leads to destabilization of the enzyme quaternary structure concomitant with dissociation into its corresponding subunits [10]. These changes can in most cases be reversed after re-warming [11,12]. Sometimes, however, cold-induced dissociation is followed by an aggregation step, which causes the irreversibility of the process [13-16]. It is believed that at low temperatures the entropic term for association becomes less favorable since the solvent molecules released upon association are more ordered. In general, the models for cold inactivation predict that the pivotal role in cold inactivation is played by hydrophobic interactions [17], but direct experimental evidence is lacking.

**Figure 1:**
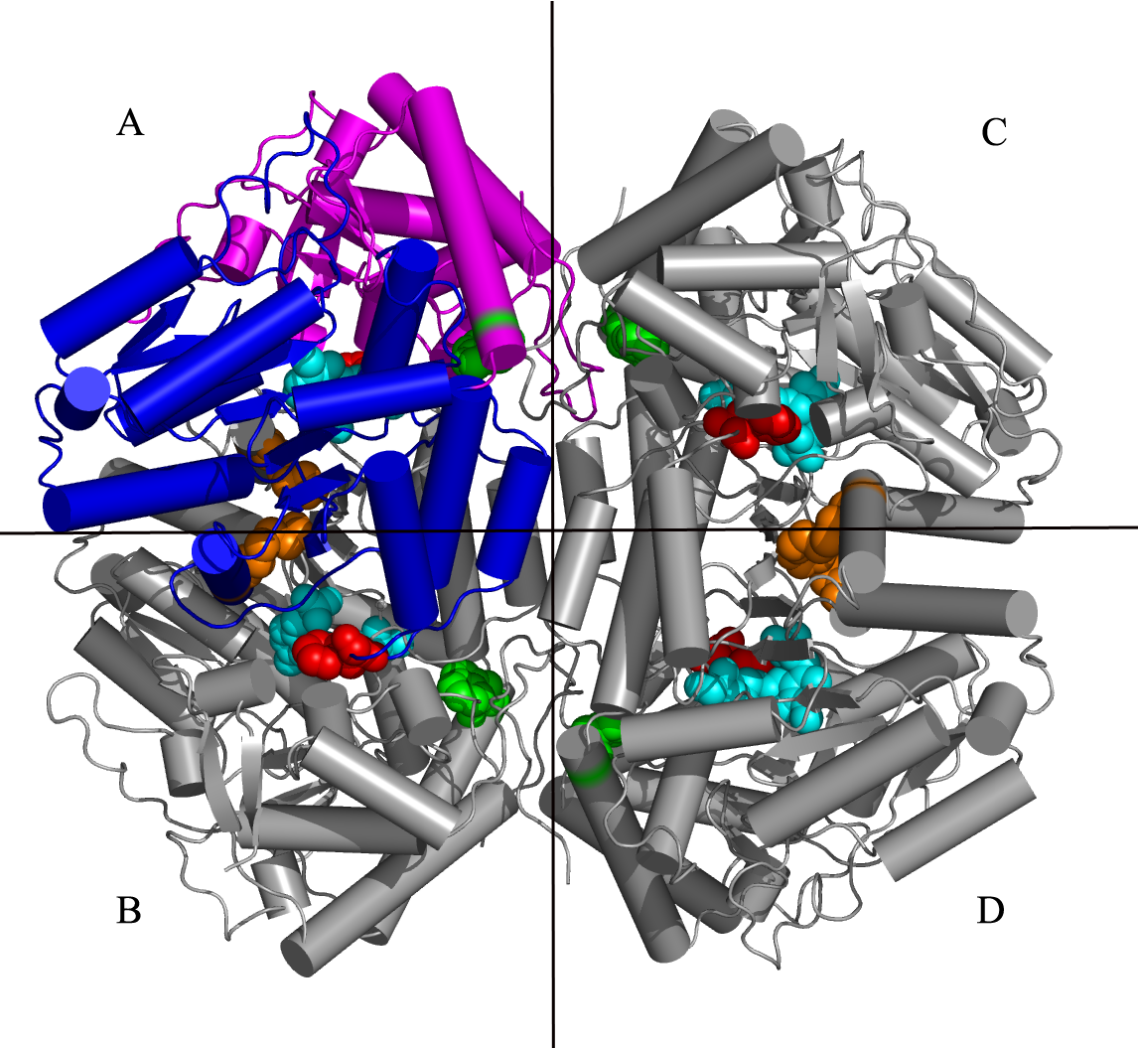
**The crystal structure of tetrameric form Trpase of *E. coli *created by symmetry operation of single monomer**. Molecule A is colored by domains; large domain is colored in blue and small domain in magenta. The other three monomers of the tetramer are colored in gray. The non-catalytic axis (vertical) and catalytic axis (horizontal) are shown as lines perpendicular to each other. Mutation sites of residue 74 (orange), residue 298 (red), and 330 (green) as well as the PLP molecule (cyane) are shown as CPK.

*E. coli *tryptophanase (tryptophan indole-lyase, Trpase, EC 4.1.99.1) is a pyridoxal 5'-phosphate (PLP)-dependent enzyme that catalyses α,β-elimination and β-replacement reactions of L-tryptophan. The molecular weight of Trpase from different species ranges from 200 to 220 kD. Trpase consists of four identical subunits (~52 kD per monomer), each of which covalently binds one molecule of PLP. The tetramer possesses a D2 symmetry, therefore it is a dimer of dimers with two different dimeric interfaces: a catalytic interface and a non-catalytic interface (Figure 1) [18-26].

Wild-type (wt) *E. coli *Trpase in its holo form undergoes a very slow and reversible inactivation after incubation at 2°C for several hours. The catalytic activity can be restored by incubation at room temperature or 37°C for several hours [11,12]. Using kinetic spectrophotometric methods and size-exclusion chromatography we have previously shown that the inactivation process is linked to the cleavage of the PLP-Lys270 aldimine bond and to the dissociation of the tetrameric form of Trpase into dimers [27]. Interestingly, the dissociation of *E. coli *Trpase never proceeds beyond dimers. It was suggested that the cooling of wt Trpase and its mutants has two effects - it weakens hydrophobic interactions and it causes the release of PLP from the active site. In order to identify which of these processes most contributes to the cold lability of *E. coli *Trpase we studied cold and high pressure inactivation, cold dissociation and the crystal structure of several Trpase variants and mutants. The mutation sites are located in different interfaces of the tetramer (i.e., the catalytic and the non-catalytic interfaces [18]) and were originally designed for understanding the contribution of each mutation to the assembly into the catalytic active tetramer.

The crystal structure of holo Trpase isolated from *Proteus vulgaris *[18] and of another two crystal structures of *E. coli *Trpases in their apo form were determined [28,29]. Both crystal structures of apo *E. coli *Trpases share the same fold as the holo *P. vulgaris *Trpase, but structural alignment of the three crystal structures revealed significant differences in the relative orientation of the two domains of the enzyme [29]. We compared these known crystal structures to the two new crystal structures of Trpase mutants we determined in order to gain structural insights into the cold-induced conformational changes and PLP release. In addition, we sought structural themes to account for the stability of *E. coli *dimers which do not dissociate into monomers as do those from *P. vulgaris*.

## Results

### Enzymatic activity measurements

Subsequent to incubation at 2°C, wt Trpase lost about 35% of its activity while the W330F and C298S mutants lost about 90% of their activity (Table 1). The Y74F mutant has low activity at 25°C and its residual activity was further reduced by cooling (Table 1).

**Table 1:** Specific activity of *wt*-Trpase and its mutants at 25°C and after cooling to 2°C in Tricine -KCl buffer, pH = 7.5.

**Trpase**	**Specific activity** **25°C**	**(Units/mg)** **2°C**
wt	45.7 ± 2.5	29.7 ± 1.7
W330F	42.8 ± 3.2	5.0 ± 1.4
C298S	36.9 ± 2.9	3.7 ± 0.8
Y74F*	0.2 ± 0.05	0.08 ± 0.03

*The concentrations of the wt Trpase and of W330F, C298S mutants were 0.3-0.5 mg/ml, while the concentration of the Y74F mutant was 10-20 mg/ml.

The cold inactivation of the W330F and C298S mutants was similar, about 90%, which is much higher than that of the wt Trpase (only 35%). Residue 330 of *E. coli *Trpase is located at the non-catalytic interface while residue 298 is located at the catalytic interface (Figure 1), yet both mutants showed a similar loss of activity. Due to the opposite change in hydrophobicity caused by the corresponding mutations (Table 2), the observed loss of activity cannot be attributed to a more hydrophobic residue [30] at the different interfaces. A plausible explanation for the loss in activity is the change in the size of the side chains of the amino acids. In all of these mutations sites the new amino acid is smaller than the wt, and the mutation enlarges the space for the side chain orientation and increases the thermal motion in the respective regions, Table 2[31-33] and references within. These mutations prevent the tight assembly of the tetramer and result in dissociation into dimers, which leads to the observed loss in activity. Although the reduction in size is relatively minor, the effect is four fold amplified since Trpase is a tetrameric enzyme.

**Table 2:** A summary of physical properties of residues affecting cold lability.

**Residue name**	**Volume (Å^3^)**	**Hydrophobicity **[55]	**Location**	**Comments**
Tyr74	193.6	-1.3	On the catalytic interface	Involved in hydrogen bonding with PLP and Arg103 from adjacent subunit
Phe74	189.9	2.8	On the catalytic interface	
Trp330	227.8	-0.9	On the non-catalytic interface	
Cys298	108.5	2.5	Close to the catalytic interface	Oxidized with 2-mercaptoethanol
Ser 298	89.0	-0.8	Close to the catalytic interface	
Val15	140.0	4.2	On the non-catalytic interface	Involved in inter subunit β-sheet network
Ile16	166.7	4.5	On the non-catalytic interface	Involved in inter subunit β-sheet network
Met 57*	162.9	1.9	On the non-catalytic interface	Involved in inter subunit β-sheet network

* Accordingly *to P. Vulgaris *numbering

In the Y74F mutant, the observed loss of activity at 25°C is presumably attributed to the proximity of residue 74 to the substrate binding site [34]. In the wt Trpase the hydroxyl group of Tyr74 is hydrogen bonded to both Arg103 (at a 2.9 Å distance), and to the phosphate group of PLP (of the neighbouring subunit in the catalytic dimer) through a bridging water molecule. Replacing Y74 by phenylalanine eliminated the hydrogen bonds of the hydroxyl group thereby reducing its affinity towards PLP. Hence, the Y74F mutant exhibited negligible activity compared to the other Trpases.

### Time dependence profile of cold inactivation and dissociation

**Figure 2:**
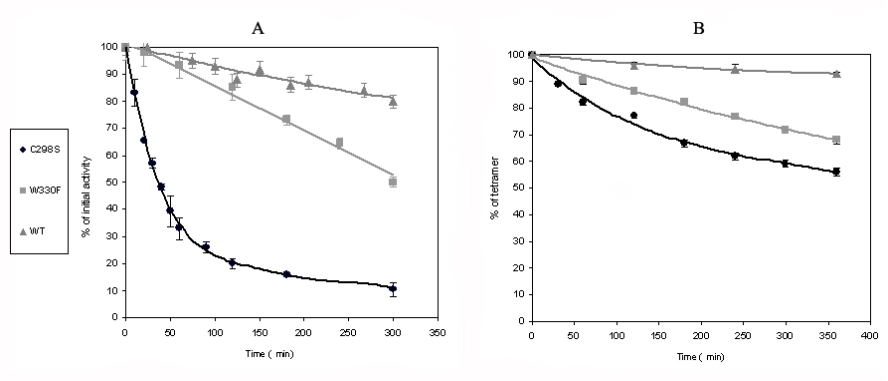
**Time dependence of cold inactivation and dissociation of wt Trpase and C298S and W330F mutants incubated at 2°C in Tricine-KCl buffer at pH 7.5**. Activity was measured by a spectrophotometric method with the chromogenic substrate analog, S-(*o*-nitrophenyl)-L-cysteine (SOPC); Protein concentration: 1.0 mg/ml.

The time course of cold inactivation of wt Trpase and its W330F and C298S mutants in Tricine-KCl buffer, pH 7.5 in the presence 0.05 mM PLP is shown in Figure 2. For C298S cold inactivation occurred much faster than that for W330F and wt Trpase. The rate constants of the initial linear phase are (13.8 ± 0.3) × 10^-3 ^min^-1^, (1.7 ± 0.1) × 10^-3 ^min^-1 ^and (0.6 ± 0.3) × 10^-3 ^min^-1 ^for C298S, W330F and wt Trpase, respectively. Both W330F and C298S exhibit similar (90%) total cold inactivation, but the time profile (Figure 2A) revealed a factor of about 8 fold faster kinetics for C298S.

The cold dissociation time profiles revealed the same pattern, with the following initial rate constants: (3.2 ± 0.1) × 10^-3 ^min^-1 ^(0.84 ± 0.1) × 10^-3 ^min^-1 ^and (0.42 ± 0.1) × 10^-3 ^min^-1 ^for C298S, W330F and wt Trpase, respectively (Figure 2B). The rate constants for cold inactivation and dissociation are similar for the wt and the W330F mutant, but in the case of C298S there is a factor of ~4 between them. This may be attributed to the relative proximity of residue 298 to the PLP and substrate binding sites as well as to its location on the catalytic interface, in contrast to that of the 330 site. The PLP in the C298S mutant is loosely bound allowing, relative to W330F and the wt, a quicker cold inactivation and dissociation.

### Dissociation of *E. coli *Trpases to dimers

The HPLC analyses of *E. coli *Trpase dissociation into dimers are presented in Table 3. The holo wt Trpase and its mutants C298S and W330F at 25°C are tetramers and upon cooling dissociate into dimers: 20% dissociation took place with the wt Trpase, whereas 60-70% dissociation took place for the three mutants. It should be noted that mutant Y74F dissociates even at 25°C (about 17%). Unless the concentration of PLP is higher than 5 mM, Y74F exists as a mixture of holo and apo forms which affects the dimer-tetramer equilibrium.

**Table 3:** The effect of cooling on the dissociation of wt Trpase and its mutants to dimers.

	**Degree of dissociation to dimers (%)**	
***E. coli *Trpase**	**25°C**	**2°C**
wt *holo*	<3	20 ± 1
C298S *holo*	<5	64 ± 1
W330F *holo*	<5	61 ± 2
Y74F *holo*	17 ± 3	66 ± 4
wt *apo*	70 ± 5	87 ± 3
C298S *apo*	>80	88 ± 1
W330F *apo*	69 ± 4	90 ± 1
Y74F *apo*	80 ± 1	83 ± 2

For the holo forms, the PLP concentration in Tricine-KCl buffer is 0.1 mM.

In contrast to the holo forms, all apo forms of Trpase markedly dissociate to dimers, about 70% at 25°C and about 90% upon cooling to 2°C suggesting that PLP has a major effect on the stability of the holo form. Once the PLP molecule is removed, the differences in the extent of dissociation among the wt and the mutants are very small. However, in the holo forms in which PLP is present, the differences between the mutants stem primarily from the effect of mutation, which in all three mutants presumably cause a reduced fit between the dimers relative to the wt form. Thus, the apo form of all Trpases showed a similar lack of stability towards cooling, indicating that the expulsion of PLP renders the apo form equally susceptible to a very pronounced cold driven dissociation.

### High pressure studies

**Figure 3:**
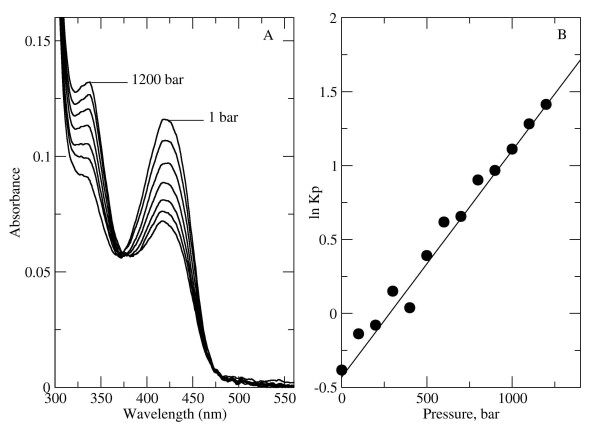
**Effect of hydrostatic pressure on the absorption spectra of wt *E. coli *Trpase (Figure 3A)**. Plot of the calculated K_p _determined from the absorbance versus the applied hydrostatic pressure (Figure 3B).

High pressure studies with wt *E. coli *Trpase revealed a rapid reversible conformational change in which the enzyme exhibits an absorption spectrum with two peaks, one at 338 nm and the other at 420 nm, corresponding to inactive and active forms of the cofactor, respectively (Figure 3). The 420 nm form of the enzyme decreases as the pressure is raised, with a concomitant increase in the 338 nm form, and there is a reasonably good isosbestic point at 374 nm (Figure 3A), indicating that at equilibrium there are two predominant species. The calculated K_p _shows the expected logarithmic relationship with hydrostatic pressure (Figure 3B), with a K_0 _(the equilibrium constant involving the two spectroscopically distinctive species with λ_max _at 420 and 338 nm, at 1 bar) of 0.65 ± 0.03 and ΔVo = -38 ± 3 mL/mol (where ΔVo is the volume difference associated with the induced conformational change). These data suggest that there is a conformational change associated with the transition between the two absorption peaks, since the relatively large negative value of ΔVo is likely a result of solvation of the exposed protein surface. It is proposed that these two spectroscopic forms of Trpase correspond to the open (338 nm) and closed (420 nm) conformations [35,36].

Hydrostatic pressure causes unfolding of proteins because there is a decrease in the volume of the protein-solvent system upon unfolding [37]. Generally, three factors are considered to contribute to ΔV_o_: electrostriction of charged and polar groups that became exposed to solvent upon unfolding or conformational change; collapse of packing defects and internal cavities; volume effects transfer of hydrophobic groups from the protein interior to water. The two first factors provide a negative contribution to ΔV_o_. However, the third factor, the exposure of hydrophobic groups to solvent can give both negative and positive contributions for changes in hydration volumes, depending on pressure and temperature [38].

In the case of the wt Trpase, the conformational change from a closed to an open conformation under increasing pressure is accompanied by large negative value of ΔV_o_. We used the Voronya [39] tool for analyzing the packing of the two crystal structures of wt Trpase which are found in the different conformations (for closed conformation we used pdb entry 2C44; and for the open conformation pdb entry 2OQX). The cavities analysis did not reveal any significant difference between the two conformations. Therefore, the collapse of internal cavities can not be the reason for the large negative value of ΔV_o_. Presumably, the main attributing factor to the large decrease in the volume is hydration of both polar and hydrophobic groups in the catalytic interface, which became exposed to water in the open conformation. At moderate pressures, the unfolding is reversible, but at pressures above 2.5 Kbar, subunit dissociation and irreversible denaturation are frequently observed.

### The overall crystal structure of Y74F and C298S

Preliminary crystallographic characterization of the crystals of the two mutants using the HKL2000 [40] program package revealed that the unit cell is 118 Å, 120 Å and 171 Å and the space group is F222, similar to the apo wt Trpase [PDB: 2OQX]. Data collection and refinement statistics for the apo Y74F and C298S mutants are summarized in Table 4. The crystal structures of the two mutants indicated that these Trpases are essentially identical and that the root mean square (RMS) of each of them with the apo Trpase [PDB: 2OQX] is about 0.3 Å. Efforts to crystallize the holo form of the wt Trpase and the Y74F and C298S mutants resulted in a low diffracting crystals. Analysis of the low resolution data revealed that these holo Trpases are found in two conformations suggesting low affinity to PLP.

**Table 4:** Data collection and refinement statistics* for Y74F and C298S mutants of *E. coli *Trpase.

	**Y74F **[PDB:2V1P]	**C298S **[pdb:2V0Y]
**Data collection**		
Space group	F222	F222
Wavelength	1.5Å	1.5Å
Unit cell	a = 118.7Å	a = 120.5Å
	b = 120.2Å	b = 118.8Å
	c = 171.7Å	c = 171.5Å
	α = 90°	α = 90°
	β = 90°	β = 90°
	γ = 90°	γ = 90°
Resolution	500.0-1.90 Å	500.0-1.98 Å
Highest resolution shell	1.97-1.90 Å	2. 05-1.98 Å
Total number of measured reflections Total number of independent reflections	155,699 47,181	186,657 42,496
Completeness (%)	97.6% (97.8%)	99.3% (96.5%)
Rsym	0.054 (0.410)	0.117 (0.456)
I/σ (I)	19.0 (2.9)	12.7 (2.9)
		
**Refinement**		
Resolution range (Å)	6.0-1.9	8.0-2.0
R (%)	19.14	21.48
R_free _(%)	22.72	22.09
Protein atoms	3682	3678
Solvent atoms	595	519
Mg^2+ ^atoms	1	1
Cl^- ^atoms	1	1
		
**RMS deviations from restraints target value**		
Bond length (Å)	0.005	0.006
Bond Angle (°)	1.21	1.22
Average B factor (for protein atoms) (Å^2^)	28.8	28.9

Data in parenthesis correspond to the high resolution shell.

**Figure 4:**
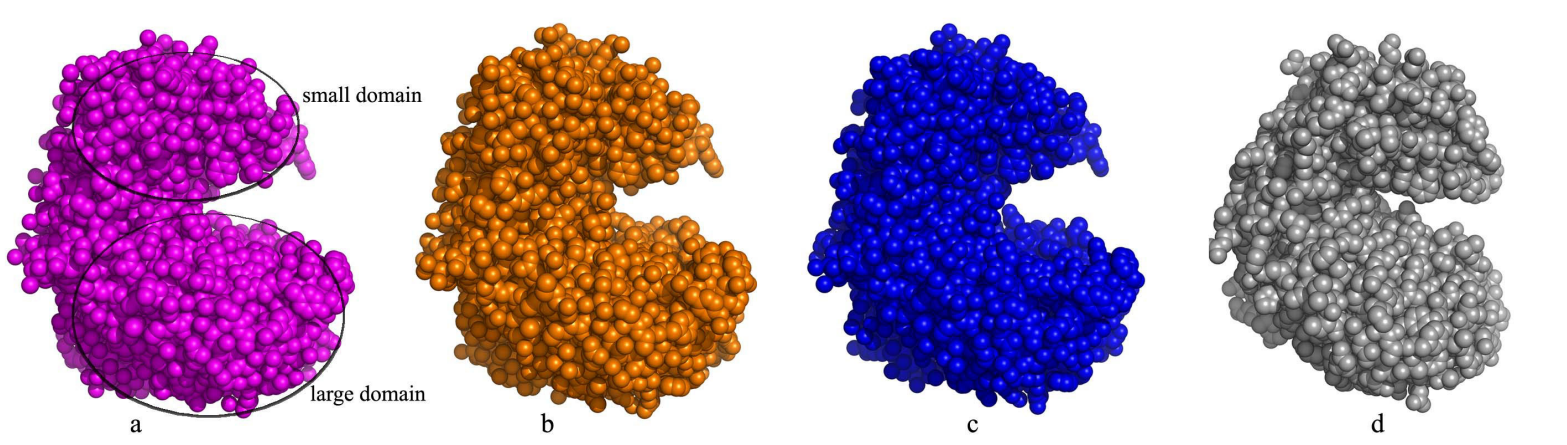
**CPK presentation of the crystal structures of apo *E. coli *Trpases and holo *P. vulgaris *(a) mutant C298S (shown in magenta), (b) mutant Y74F (shown in orange), (c) apo wt *E. coli *(shown in blue) and (d) holo *P. vulgaris *Trpase (gray)**. This indicates that the two apo mutants of *E. coli *Trpases as well as the wt Trpase are found in an open conformation, different from the closed conformation found for *P. vulgaris *in its holo form.

Amino acid sequence comparison of *E. coli *Trpase with the *P. vulgaris *Trpase indicated that these two enzymes share a 52% sequence identity [41], resulting in a similar crystal structure. However, superpositioning of the crystal structures of the wt apo *E. coli *Trpase, the two new mutants in their apo form and the holo *P. vulgaris *Trpase revealed significant differences in the relative orientation of the large and small domains (Figure 4). The two apo mutants of *E. coli *Trpases as well as the wt Trpase are found in an open conformation, different than that of the closed one found for *P. vulgaris *in its holo form. The RMS deviation for 464 Cás of the C298S mutant crystal structure with the Trpase from *P. vulgaris *(of subunit A) is 4.8 Å, while the RMS deviation for each domain is 2.1 Å for the large domain (for 260 Cαs) and 1.1 Å for the small domain (for 152 Cαs of the small domain).

**Figure 5:**
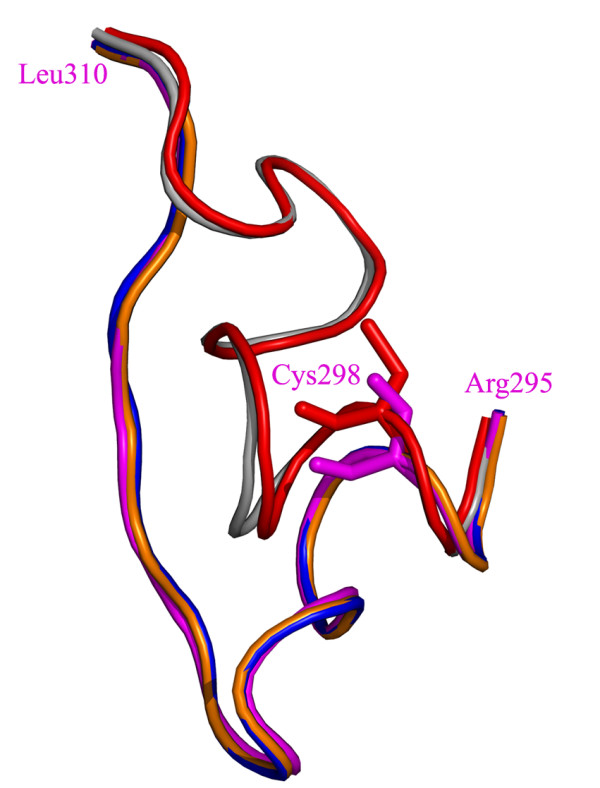
**Different conformations of the loop composed of residue 295-310 (*E. coli *numbering)**. The apo wt *E. coli *loop is colored in blue, the mutant Y74F is colored in orange and C298S in magenta. The same region of holo *P. vulgaris *Trpase is (in gray) and apo wt *E. coli *(in red) indicating an additional conformation for this loop. The mutation site 298 is shown as sticks for the apo *E. coli *(red) and for the C298S mutant in magenta.

Additional significant differences among the holo Trpase from *P. vulgaris *and the apo wt *E. coli *Trpase were found in the conformation of the loop composed of residues 295-310 (*E. coli *numbering), and are shown in Figure 5. This region is found in two major conformations: one of the holo forms and one of the apo forms. In one of the wt apo crystal structures this loop is found in the same conformation as holo *P. vulgaris *Trpase. This suggests that the loop composed of residues 295-310 is highly flexible and may be involved in the transition between the closed and open conformation. The flexibility of this region may account for the faster kinetics found for C298S mutant [28].

**Figure 6:**
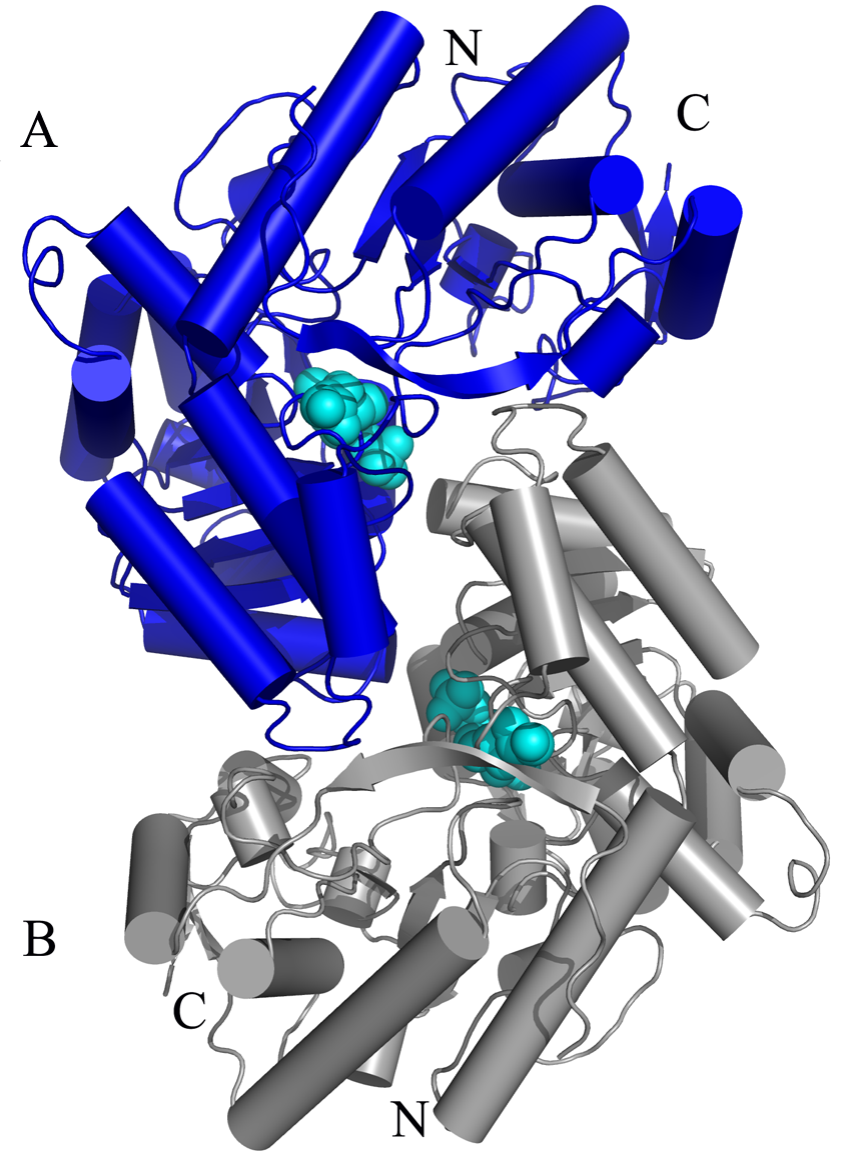
**The catalytic dimer is composed of A and B molecules**. The PLP molecule is located in the inter domain cleft of one monomer and is coordinated by residues from a neighboring monomer.

The PLP molecule is located in the inter domain cleft of one monomer and is coordinated by residues from a neighboring monomer [PDB: 1AX4] (Figure 6). The two monomers involved in the binding of the PLP molecule (subunits A and B, Figure 1) form a dimer resembling the aspartate aminotransferase (AATase) dimer [42]. This dimer is referred to as the catalytic dimer, while subunits A and C create the non-catalytic dimer (Figure 1). We have examined the interactions holding the monomers of *E. coli *Trpase along the catalytic and the non-catalytic interfaces [18]. The catalytic dimer (AB) is further stabilized by two hydrogen bonds between the hydroxyl groups of Tyr74 and Tyr307 from one subunit to the phosphate group of the PLP molecule from the neighboring subunit. Upon cooling and conformational change these hydrogen bonds are distorted and weakened.

**Figure 7:**
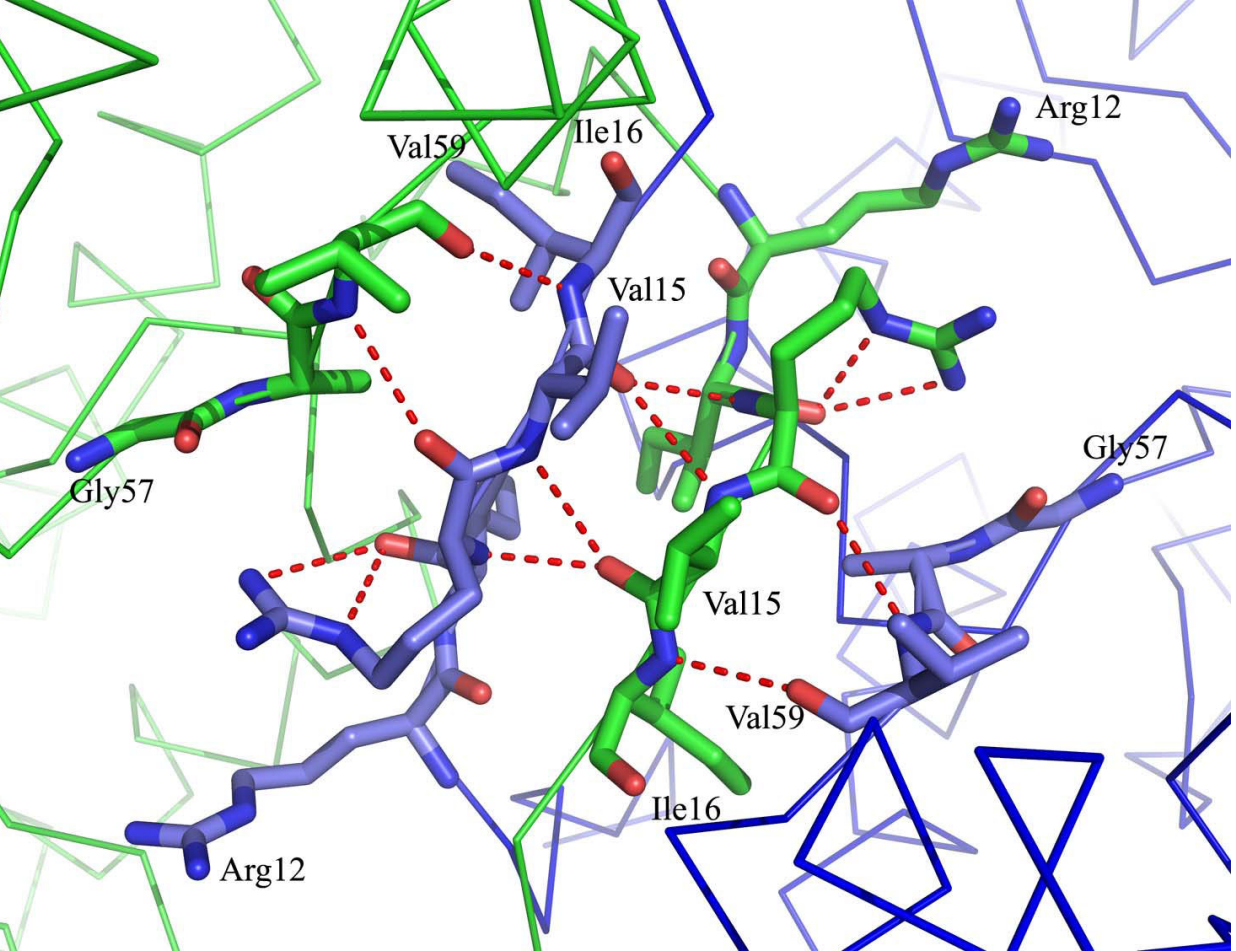
**The β sheet at the non-catalytic interface is formed by two anti parallel β strands from subunits A (shown in blue) and C (shown in green)**. This hydrophobic core is not exposed to the solvent and accounts for the dissociation of *E. coli *Trpase to dimers only.

On the non-catalytic interface there are numerous PLP-independent interactions that include the hydrogen bond network of a β-sheet formed by four β strands between subunits A and C (residues 12-17 and 58-60 from each subunit) and the hydrophobic interactions in this region include the side chains of Val15 and Val59 (Figure 7). These interactions along the non-catalytic interface are relatively far from the active site and the PLP binding site and as such are not affected by the binding or the release of PLP. Once *E. coli *Trpase undergoes a conformational change these strong hydrogen bonds and hydrophobic interaction between the two subunits prevent further dissociation of the dimers to monomers. Thus, we suggest that upon cooling, the Trpase tetramer dissociates into the non-catalytic dimers (resulting in AC and BD dimers).

### Dissociation of *P. vulgaris *Trpase

We also examined the cold dissociation of apo Trpase from *P. vulgaris *and compared it to the cold dissociation of *E. coli *Trpase. Despite the similarity in the amino acid sequence between both enzymes they show different stability at low temperatures (2°C). Trpase from *P. vulgaris *in its apo form dissociates further into monomers even at 25°C (Table 5). Cooling the *P. vulgaris *Trpase to 2°C for 24 h augmented the dissociation into monomers. This is in sharp contrast to the *E. coli *Trpase which dissociates to dimers only.

**Table 5:** The effects of cooling and reactivation on the degree of dissociation of apo wt Trpase from *E. coli *and *P. vulgaris*

**Sample**	**T (°C)**	**Degree of dissociation (%)**		
		**Tetramer**	**dimmer**	**monomer**
*P. vulgaris *Trpase	25°C	37.3 ± 1.2	44.5 ± 3.4	18.2 ± 4.0
*P. vulgaris *Trpase	2°C, 24 h	21.7 ± 0.6	45.4 ± 0.7	32.9 ± 1.6
*P. vulgaris *Trpase +1 mM PLP	25°C, 2 h	92.6 ± 0.8	7.4 ± 0.2	-
*E. coli *Trpase	25°C	30.0 ± 2.2	70.0 ± 5.0	-
*E. coli *Trpase + 1 mM PLP	25°C, 1 h	100 ± 0.6	-	-

Data in parenthesis correspond to the high resolution shell.

Analysis of the non-catalytic interface in both Trpases (from *E. coli *and *P. vulgaris*) revealed that the β-sheet located at the core of the tetramer is not exposed to the solvent. Furthermore, the four β-strands are structurally similar, but differ in their amino acid content. Met57, Met13 and Val14 of *P. vulgaris *Trpase are replaced by Val59 and Val15 and Ile16 in the *E. coli *Trpase (Table 2). The two valine residues and the isoleucine residue of *E. coli *Trpase are more hydrophobic (see table 2) than the corresponding two methionine residues and the valine residue of *P. vulgaris *[30]. Thus, the presence of the more hydrophobic core in the region of the non-catalytic interface of *E. coli *presumably leads to a higher stability of the dimeric structure of apo Trpase from *E. coli *compared to Trpase of *P. vulgaris*.

## Discussion

At low temperatures and low concentrations *E. coli *Trpase undergoes a reversible loss of activity, followed by dissociation into dimers. Complete dissociation/inactivation occurred after incubation for 20 h at 2°C and association/reactivation occurred upon warming for few hours. This was previously reported in a detailed time course study for both the wt Trpase and its W330F mutant [11,12,27]. To further explore the temperature driven changes in the hydrophobic interactions at the non-catalytic or at the catalytic interfaces of *E. coli *Trpase we examined the role of single point mutation at each interface on enzyme activity, dissociation and crystal structure.

**Scheme 1:**
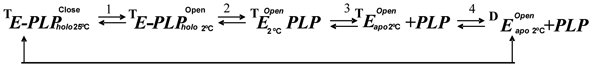


Based on our kinetic and high pressure studies as well as the crystal structures, we propose the following mechanism for the cold inactivation of *E. coli *Trpase (Scheme 1)

where ^T^E-PLP^close ^is the holo tetrameric active Trpase at 25°C in a close conformation; ^T^E-PLP^open ^is the holo tetrameric enzyme in the open conformation at 2°C; ^T^E^... ^PLP is the non-covalently bound complex in an open conformation and ^T^E is the apo enzyme in an open conformation at 2°C. Step 4 is the dissociation of the tetrameric form to dimers, ^T^E to ^D^E.

Cooling (step 1) weakens the hydrophobic interactions resulting in a conformational change and a corresponding modified solvation. The change from the closed to an open conformation is associated with a reduction in the tight packing of the tetramer and is enhanced by the point mutations. This conformational change is further supported by the present high pressure studies showing that increasing hydrostatic pressure has the same effect as decreasing temperature in affecting the conformational equilibrium.

The conformational change at low temperatures results in breaking the covalent aldimine bond between residue Lys270 (*E. coli *numbering) and the PLP molecule (step 2). This overcomes the rest of the non-covalent binding interactions between PLP and Trpase, and results in the separation of the PLP from the active site (step 3) leading to dissociation into dimers (step 4). This suggested sequence for PLP-Trpase interactions is further supported by HPLC-Diode array profiles which could not detect PLP-bound dimers.

Our results suggest that even minor changes in the size of the amino acid side chain reduce the tight fit that is presumably required for the stability of the holo tetrameric enzyme. The reduced fit due to mutations along the two symmetry axis resulted in a higher vulnerability to cooling, which enhances the dissociation and the release of PLP. Changes in side chain hydrophobicity in the present mutants were found to be less relevant to the stability of the tetramer.

We also compared the dissociation of the wt and the three mutants in their apo form and found that their dissociation into dimers occurs even at 25°C to the same extent (Table 3). Upon cooling of the apo forms to 2°C, an additional dissociation of about 15% was observed. The effect of the point mutation on the cold dissociation was observed only in the holo form. We found that at 2°C, a factor of 3 in the extent of dissociation was observed between the wt and its mutated holo forms (Table 3). The loss of PLP overshadows the relative small contributions of the different interactions in the mutants, suggesting that these interactions at both interfaces have a relatively minor effect on the cold dissociation of *E. coli *Trpase.

In contrast to the similarity found in the degree of cold inactivation, dissociation (Tables 1&3) and the crystal structures (Figure 5), differences were observed in the kinetics of cold inactivation and dissociation between the mutants and the wt Trpase (Figure 2). The location of the point mutation, i.e. on the catalytic (C298S) *vs*. the non-catalytic (W330F) axis, affects primarily the kinetics of cold lability. Notably, while a qualitative correlation was observed between the kinetics of cold inactivation and dissociation, it does not follow a quantitative relationship. While cold inactivation is a direct result of primarily the enamine PLP-Lys270 scission, the following collapse of the quaternary structure into dimers results from numerous protein-protein and protein-solvent interactions including hydrophobic, electrostatic and hydrogen bonds. A detailed description of this multi-step process requires the study of additional point mutations.

To summarize, the observed cold lability of *E. coli *Trpase results from the release of PLP and from the preclusion of the tight assembly of the tetramer. The wild-type system is balanced to favor the tetramer stabilization, and even a small perturbation of the interaction energy causes it to shift toward dimers.

## Conclusion

The cold inactivation and dissociation of holo *E. coli *Trpase is mainly affected by the release of the cofactor PLP. In the absence of PLP variable packing-interactions on the monomers' interface due to the mutation (Y74F, C298S and W330F) make additional, relatively small contribution to the cold dissociation of *E. coli *Trpase to dimers. The hydrophobic interactions along the non-catalytic interface may account for the difference in dissociation into dimers *vs*. monomers in *E. coli *and *P. vulgaris *Trpases.

## Methods

### Materials

S-(*o*-nitrophenyl)-L-cysteine (SOPC) was synthesized as described previously [43]. PLP, Tricine, L-tryptophan, 2-mercaptoethanol, protamine sulfate, ampicillin, buffers and (NH_4_)_2_SO_4 _were purchased from Sigma. Other chemicals obtained from various commercial suppliers were of pure or extra pure grade.

### Enzyme purification

Wild-type Trpase was isolated from *E. coli *SVS 370 cells containing the *tnaA *gene on plasmids by a procedure described previously [44]. Additional purification was achieved by anion exchange chromatography on DEAE-Sephadex A-50 or DEAE-Sepharose 4B-CL, and size exclusion chromatography on Sephacryl S-300-HR [11,45]. The protein fractions with the highest specific activity and homogeneity in SDS polyacrylamide gel electrophoresis (greater than 97% purity) were combined and concentrated by total precipitation with (NH_4_)_2_SO_4 _or in an Amicon ultrafiltration YM-30 membrane and frozen in 0.3-ml aliquots at -80°C. W330F and C298S mutants of *E. coli *Trpase were isolated in the same way from *E. coli *SVS 370 cells containing the *tnaA *gene with the respective site-directed mutation. The Y74F mutant was expressed under lac., on a pET17b plasmid in BL21(DE3) cells. Wild-type Trpase from *P. vulgaris *was isolated from *E. coli *SVS 370 cells containing the tryptophanase gene from *P. vulgaris *in the pAVK plasmid [41]. Protein concentration during purification was determined using the Bradford reagent [46] with BSA as a standard. The concentration of each purified enzyme (wt, C298S and Y74F) was determined at 278 nm, taking the absorbance A^1% ^values of 9.19 for holo *P. vulgaris *and holo *E. coli *and 7.95 for apo *wt*-Trpases [44]. The concentration of W330F was determined using the value of 7.64 for the holo form [47]. The apo enzymes were prepared by overnight dialysis of the holo enzymes against 0.1 M sodium phosphate-ethylenediamine buffer, pH 8.0, at 4°C.

### Enzymatic activity measurements

Activity was measured by a spectrophotometric method with the chromogenic substrate analog, S-(*o*-nitrophenyl)-L-cysteine (SOPC), as described in Suelter *et al*., [21,48] using the 8453A Hewlett Packard spectrophotometer connected to a UC-F-10 Julabo thermostated bath (JTB) (± 0.1°C). A-10 μl enzyme aliquots (0.3-0.5 mg protein/ml, specific activity 42-50 μmol min^-1^mg protein^-1^) were stirred into 1 ml (final volume) of 0.6 mM SOPC in 50 mM potassium phosphate buffer, pH 8.0, at 25°C and. Initial activity was measured for 1 min by following the decline in OD_370 nm _(Δε = -1.86 × 10^3 ^M^-1^cm^-1^). One unit of Trpase is defined as the amount required for the decomposition of 1 μmol SOPC to the product *o*-nitrothiophenolate in 1 min at 25°C. The activity measurements related to cold inactivation were carried out as described above. The activities of the Trpases at 2°C were measured within 30 to 60 sec, a time interval which is short enough to prevent reversibility of the cold-inactivated form. We previously have shown that the reactivation requires a minimum of 1 h of re-warming to 25°C [11,12,27,49]. The concentrations of the wt Trpase and of W330F, C298S mutants were 0.3-0.5 mg/ml, while the concentration of the Y74F mutant was 10-20 mg/ml [50].

### Time dependence of cold inactivation and dissociation

The time dependence of cold inactivation of wt Trpase and its mutants were performed at 25°C in 50 mM Tricine-KOH buffer at pH 7.5 containing 100 mM KCl (Tricine-KCl), 5 mM 2-mercaptoethanol, 50 μM PLP. The enzyme solution (0.5-1 ml) was kept on ice and slightly stirred. Aliquots of 10-20 μl were removed at various times for assay at 25°C. The time profile of cold dissociation for the wt, C298S and W330F was followed by HPLC analysis as described below.

### HPLC measurements of dissociation degree for holo and apo Trpases

HPLC analysis of Trpases was performed at 25°C and 2°C with a Waters 99 supplied Photodiode Array detector UV using a gel filtration column BIOSEP-SEC S-3000 (7.5 × 600 mm), equipped with a water jacket connected to a thermostated bath (UC-F-10 Julabo). Cold dissociation studies were carried out by incubating the enzymes for 20 h at 2°C at a concentration of 1 mg/ml in Tricine-KCl, 2 mM EDTA, 5 mM 2-mercaptoethanol and 50 μM PLP. The HPLC column was loaded with 20 μl of Trpase in Tricine-KCl. The HPLC-chromatogram showed two peaks. The first displayed a PLP-bound characteristic absorption spectrum of the tetrameric form with maxima at 278, 337 and 420 nm, and the second displayed a PLP-free enzyme peak, which is the dimeric form, characterized by the absence of the 337 and 420 nm peaks. Control samples incubated at 25°C for 1 h fully preserved their tetrameric structure. All data of specific activity and degree of dissociation are presented with the associated standard errors. HPLC measurements of apo Trpases were performed in the same buffer in the absence of PLP. The HPLC chromatogram of apo Trpases revealed two peaks with maximum absorbance at 278 nm.

### High pressure studies

The effect of hydrostatic pressure on the absorption spectra measured using a Cary 14 UV-VIS spectrophotometer modified by OLIS, Inc., containing a high-pressure cell from ISS (Champaign, IL) and equipped with a manual pressure pump. The cell was maintained at 25°C with an external circulating water bath. Samples (1.2 ml) containing 1 mg/ml Trpase in 0.1 M triethanolamine hydrochloride, pH 8.0, were enclosed in quartz bottles with a 9 mm path length immersed in spectroscopic ethanol as the pressurizing fluid.

The effect of pressure on the quaternary equilibrium was examined by incubating wt Trpase at elevated pressures, and following the changes in absorption spectrum as the PLP is released. Pressure affects equilibrium according to Eq. 1, where K_p _is the equilibrium constant at pressure P, K_o _is the pressure-independent value of the equilibrium constant, ΔV_o _is the reaction volume change, R is the gas constant (0.08314 l-bar/mol K) and T the temperature in Kelvin [51]. The logarithmic form of Eq. 1 is given in Eq. (2). Thus, a plot of ln Kp versus *P *will give a straight line with a slope equal to Δ*V*o/*RT *and intercept at ln K_o_.

(1)

(2)

(3)

The equilibrium constant can be related to the change in absorbance at a particular wavelength by Eq. 3 for simple two species equilibrium, where A_p _is the absorbance at pressure P, A_o _is the absorbance at 0 bar, and A_∞ _is the absorbance at infinite pressure. The values of A_o_, A_∞_, K_eq _and ΔV_o _were obtained by global fitting of the spectra at all wavelengths from 300 to 550 nm throughout the entire range of pressures to Eq. 1 and 3 using the GlobalWorks program provided by OLIS, Inc. [52]. The protein concentration would be expected to increase with pressure because of the compressibility of water, which would result in a maximum increase in concentration of 4.7% at 1200 bar. However, the spectra were not corrected for the pressure effect, and the experimental results nonetheless fit well to the model, with low standard errors. The data also show an isosbestic point in the spectra, indicating that the concentration change due to pressure does not significantly affect the results.

### Crystallization of Y74F and C298S

Crystallization experiments were carried out at 20°C, and were set up using the hanging-drop vapor-diffusion method with siliconized cover-slips and Linbro 24-well tissue culture plates. Droplets ranging in size from 5 to 10 μl prepared by mixing equal volume of the protein solution and the reservoir solution and were equilibrated with 1.0 ml of reservoir solution at room temperature (20°C). The protein at concentration of 30-50 mg/ml in 50 mM Tris, 100 mM KCl pH 7.5, 2 mM EDTA and 5 mM 2-mercaptoethanol was mixed with the reservoir solution containing 30%(w/v) PEG 400, 100 mM HEPES pH 7.5, 200 mM MgCl_2_, 5 mM 2-mercaptoethanol [45].

### Data collection and structure determination

Crystals were transferred to Paratone oil (Hampton Research) and excess liquid was removed, followed by freezing in liquid nitrogen. Diffraction data set for the apo Y74F mutant was collected using a Rigaku RU3H rotating anode generator and MAR345 image plate detector at 100 K. For the apo C298S the diffraction data set was collected at low temperature (100 K) using a Raixs IV electronic area detector and a Rigaku RU-200 HB generator. Unit cell parameters, crystal orientation and integration of reflection intensities were determined using the HKL2000 package [40]. Refinement of the data was done using CNS [53] and manual corrections of the model were carried out with the graphics program O [54].

## Authors' contributions

AK crystallized the mutants and determined their crystal structures. GYG isolated the enzymes and perform the HPLC studies. RCL participated in the kinetic data analysis and helped to draft the manuscript. YG participated in the crystal structure determination and analysis. RSP carried out the high pressure study and prepared the various mutants. AHP conceived of the study, participated in its design and coordination as well as helped to draft the manuscript. OA made substantial contributions to the design of the study, acquisition of crystallographic data, analysis and interpretation and writing the manuscript. All authors read and approved the final manuscript.
